# Paraffin Injections

**Published:** 1902-06

**Authors:** 


### TRANSLATION.

### Paraffin Injections.

Translated by HERMAN MYNTER, M. D., Buffalo, N. Y.

From *Hospitals-Tidende.*

WE HAVE probably all read about the paraffin injections of Gersuny, of Austria, and how he has succeeded in remedying different defects by subcutaneous injection of paraffin, it having been shown that paraffin of a melting point a little higher than the normal temperature of the blood, remains indefinitely without reaction and may be shaped to overcome certain cosmetic defects. Gersuny in that way substituted in one case paraffin testes for the testes removed by castration; in another case overcame incontinence of urine in a woman with widely dilated urethra with prolapse of the mucous membrane by submucous injections of paraffin.

Cases of cystocele, prolapse of vagina, and prolapse of uterus have been successfully treated by injections of paraffin into the vaginal wall and parametrium. Also, in inguinal hernia it has been used, the canal being considerably narrowed by its effect. Unsightly deformities of the face have been corrected with startling results, such as the deformity resulting from resections of the superior maxilla, saddle-nose, pits after smallpox, and the like.

The paraffin should have a melting point slightly above the normal temperature of the body, between 99° and 104°. If below the body temperature it will soon be carried away by the lymphatics, and if too hard, it may produce neuroses. Dr. Rupert M. Parker, of Chicago, from whom I largely quote (see *Journal American Medical Association*, April 19, 1902,) gives the following rules for the preparation of the paraffin: the ordinary lump paraffin is much too hard, and the paraffin known as medicinal vaseline too soft. These two products may be melted together in proportions to secure the desired consistency. The melting point may be tested with the clinical thermometer, the bulb being smeared with a thin coating of the wax and immersed in a water bath, which is gradually heated until the melting point is reached. Then the wax loosens itself and floats to the surface, the thermometer indicating the temperature. Parker's paraffin melts at 102°, has the consistency of vaseline and should be sterilised by heating before use.

The paraffin is injected by an ordinary hypodermic syringe with a large needle, the syringe being aspirated full of the melted paraffin and placed in hot water until needed. No anesthesia is necessary; or at most Schleich's method may be used. The paraffin is allowed to cool sufficiently to flow from the needle as a wormlike, semisolid, coherent string. The paraffin is distributed as desired by changing the position of the needle-point, while an assistant with the fingers molds it into the desired shape. Parker reports two cases with photographs of saddle-noses, in which the result seems simply perfect; the paraffin gradually increasing in hardness until the consistency of cartilage is reached. The only danger seems to be embolism of the lung if a vein should happen to be injured by the needle.

The following curious case reported in *Hospitals-Tidende*, of April 2, 1902, by Dr. Holger Trautner, in Grenaa, Denmark, is of unusual interest:

A young workman, 20 years of age, consulted the author on March 27, 1901. Nine months previously, in June, 1900, he suffered from acute mental disturbances, probably produced by a sunstroke, during which he completely castrated himself. It was impossible for him to explain why he did it or how he did it; he had never been insane or suffered from mental symptoms. He went to Dr. Trautner because he was in despair on account of the loss of his testes; he thought that every one knew all about it and despised him on that account. He asked the doctor if he could not do something for him. "Do you mean, can I make you a new pair of testes?" "Yes, if it is possible I would be very happy." "Yes, why not?"

After thorough sterilisation and disinfection the doctor thereafter injected 30 grams pure vaseline into each half of the scrotum. The operation was done at his office and without anesthesia, for secrecy's sake. The vaseline got stiff quickly and had the form of testes. The operation was not particularly painful. The patient returned to his home, walking four miles, and suffered no bad consequences. Since that time he had had no trouble with his new testes; and the doctor learned, through the patient's family, that he had been happy and satisfied. He took care to let it be known, little by little, that the castration had not been so perfect as rumor said, that he was just as good a man as anybody else and it is probable that he occasionally exposed himself in order to prove it. He worked the whole summer and was perfectly well.

On January 14, 1902, ten months after the vaseline injection, he again called on Dr. Trautner and asked for a certificate to show in court, he having been sued for bastardy. He was very sorry to have the facts known, but unwilling to run the risk of being adjudged guilty and forced, according to Danish law, to pay eight crowns a month for eighteen years for the support of the child, so much the more as the girl seemed to have a good case, as he himself had bragged of having had intercourse with her.

The doctor gave him the following certificate: "N.N., is incapable of having sexual intercourse resulting in conception on account of total loss of his testes, the testes he has being formed artificially." He was therefore adjudged innocent, the girl, however, denying the justice of the decree.

The patient stated, furthermore, that he was able to have intercourse and that he had a sensation of ejaculation, although he knew this did not take place. He was, however, so impressed with the resources of medical science that he earnestly asked Dr. Trautner to give him something "which would make him perfectly normal again."

In the following number of *Hospitals-Tidende*, April 9, 1902, Dr. Jahrtmann pertinently asks who was the father of the child, and objects to Dr. Trautner's certificate, partly on the ground that a man may have more than two testes, cases being known in which, besides the normal testes in the scrotum, two more were found in the abdominal cavity; and partly because the semen perhaps may be retained in the vesicula seminalis for a long time. In regard to the first objection, Dr. Thulstrup mentions a case in which a man was rejected as a soldier because he had a third testis in the inguinal canal. He thinks, therefore, that a man possibly may be a father even if he has two vaseline testes in his scrotum.

[Note by the Editor.—This contribution by Dr. Mynter is erroneously printed under the head of "Translation." It is a contribution to the Journal by Dr. Mynter, and is only in part a translation from *Hospitals-Tidende*, as readily will be observed by the reader.]

